# treespace: Statistical exploration of landscapes of phylogenetic trees

**DOI:** 10.1111/1755-0998.12676

**Published:** 2017-05-15

**Authors:** Thibaut Jombart, Michelle Kendall, Jacob Almagro‐Garcia, Caroline Colijn

**Affiliations:** ^1^ Department of Infectious Disease Epidemiology MRC Centre for Outbreak Analysis and Modelling School of Public Health Imperial College London London UK; ^2^ Department of Mathematics Imperial College London London UK; ^3^ Wellcome Trust Centre for Human Genetics University of Oxford Oxford UK

**Keywords:** incongruence, multivariate analysis, package, software, tree distances, tree metric

## Abstract

The increasing availability of large genomic data sets as well as the advent of Bayesian phylogenetics facilitates the investigation of phylogenetic incongruence, which can result in the impossibility of representing phylogenetic relationships using a single tree. While sometimes considered as a nuisance, phylogenetic incongruence can also reflect meaningful biological processes as well as relevant statistical uncertainty, both of which can yield valuable insights in evolutionary studies. We introduce a new tool for investigating phylogenetic incongruence through the exploration of phylogenetic tree landscapes. Our approach, implemented in the R package treespace, combines tree metrics and multivariate analysis to provide low‐dimensional representations of the topological variability in a set of trees, which can be used for identifying clusters of similar trees and group‐specific consensus phylogenies. treespace also provides a user‐friendly web interface for interactive data analysis and is integrated alongside existing standards for phylogenetics. It fills a gap in the current phylogenetics toolbox in R and will facilitate the investigation of phylogenetic results.

## 1 INTRODUCTION

Genetic sequence data are becoming an increasingly common and informative resource in a variety of fields including evolutionary biology (Wolfe & Li, 2003), ecology (Hudson, 2008), medicine (Weinshilboum, 2002) and infectious disease epidemiology (Holden et al., 2013; Pybus & Rambaut, 2009). Although specific methods emerge to tackle particular problems in different fields, many analyses of homoplasy, selection and population structure begin with a reconstructed tree. Indeed, phylogenetic reconstruction remains the gold standard for assessing the evolutionary relationships amongst a set of taxa or sampled isolates (Bouckaert et al., 2014; Popescu, Huber, & Paradis, 2012; Ronquist & Huelsenbeck, 2003; Schliep, 2011) in the absence of horizontal gene transfers and recombination events (McInerney, Cotton, & Pisani, 2008).

Ideally, a single phylogenetic tree could be used to visualize the evolutionary history of a set of sequences. In practice, however, a number of biological and statistical factors may lead to phylogenetic uncertainty and incongruence (Jeffroy, Brinkmann, Delsuc, & Philippe, 2006; Kumar, Filipski, Battistuzzi, Kosakovsky Pond, & Tamura, 2012; Som, 2015). In such cases, several phylogenies may be equally supported by the data and need to be examined. Besides horizontal gene transfers (Delsuc, Brinkmann, & Philippe, 2005; McInerney et al., 2008), genomic reassortments (Nelson et al., 2008) and gene loss and acquisition (Page & Charleston, 1997), incomplete lineage sorting can lead different genes to exhibit distinct genealogies (Jeffroy et al., 2006; Pollard, Iyer, Moses, & Eisen, 2006; Som, 2015) and invalidate the idea of a “single evolutionary history” (Jeffroy et al., 2006; McInerney et al., 2008). Statistical uncertainty in tree topology can also arise when using bootstraps (Efron 1992; Felsenstein, 1985, Newton, 1996; Soltis & Soltis, 2003) or when considering samples of trees in Bayesian approaches (Drummond & Rambaut, 2007; Huelsenbeck, Rannala, & Masly, 2000; Ronquist & Huelsenbeck, 2003).

Because examining multiple phylogenies quickly becomes impractical, this problem is classically addressed by choosing a single reference phylogeny and indicating support for individual nodes in the other trees (Drummond & Rambaut, 2007; Felsenstein, 1985; Paradis, Claude, & Strimmer, 2004; Soltis & Soltis, 2003). Unfortunately, bootstrap or posterior support values can only be easily interpreted when they show high congruence, and considerable effort has been devoted to quantifying the credibility or probability of clades in reconstructed phylogenies (Anisimova, Gil, Dufayard, Dessimoz, & Gascuel, 2011; Drummond, Ho, Phillips, & Rambaut, 2006; Holmes, 2003b; Lemey, Rambaut, Drummond, & Suchard, 2009; Newton, 1996; Wróbel, 2008). Statistically significant results derived from different data sources can differ (Kumar et al., 2012), and while this would usually result in low bootstrap values, anomalously high bootstrap values can result from concatenation of gene sequences (Gadagkar, Rosenberg, & Kumar, 2005; Kumar et al., 2012). While several different phylogenies can be nearly equally supported by the data (Wróbel, 2008), in practice these alternative often remain unexplored (Felsenstein, 1985; Holmes, 2003a; Newton, 1996). A more satisfying alternative would consist of extracting the essential differences and similarities amongst a set of trees, visualizing these relationships and identifying one or more representative trees (Amenta & Klingner, 2002; Chakerian & Holmes, 2012; Hillis, Heath, & St John, 2005; Holmes, 2003b; Nye, 2014).

Several metrics and measures of dissimilarity between trees have been developed (Table 1), each of which directly compares trees to each other according to certain biological or mathematical properties (Critchlow, Pearl, & Qian, 1996; Estabrook, McMorris, & Meacham, 1985; Hein, Jiang, Wang, & Zhang, 1996; Kendall & Colijn, 2015; Pavoine, Ollier, Pontier, & Chessel, 2008; Robinson & Foulds, 1979, 1981; Williams & Clifford, 1971). Interestingly, these methods of pairwise tree comparison can form the basis of further analyses aiming to visualize and characterize relationships in a whole set of phylogenies. Several studies have also focussed on providing Euclidean visualizations of tree spaces, but typically relied on a single tree metric (Amenta & Klingner, 2002; Chakerian & Holmes, 2012; Hillis et al., 2005; Kendall & Colijn, 2016; Wilgenbusch, Huang, & Gallivan, 2017).

**Table 1:** Methods available in treespace for defining distances between trees

Metric/tree summary	References	R function (*package*)
Robinson–Foulds metric	(Robinson & Foulds, 1979, 1981)	RF.dist (phangorn) (Schliep, 2011) dist.topo (ape) (Paradis et al., 2004)
Branch score distance	(Kuhner & Felsenstein, 1994)	KF.dist (phangorn) (Schliep, 2011)
Billera–Holmes–Vogtmann metric (BHV)	(Billera et al., 2001)	dist.multiPhylo (distory) (Chakerian & Holmes, 2013)
Path difference metric (a.k.a. patristic distance/node distance/tip distance/dissimilarity measure)	(Steel & Penny, 1993), (note also the *l* ^*1*^ *‐*norm version by [Williams & Clifford, 1971; ])	path.dist (phangorn) (Schliep, 2011) distTips (adephylo) (Jombart et al., 2010a)
Kendall–Colijn metric	(Kendall & Colijn, 2015)	treeDist (treespace)
Abouheif's dissimilarity	(Pavoine et al., 2008)	distTips (adephylo) (Jombart et al., 2010a)
Sum of direct descendents	(Pavoine et al., 2008)	distTips (adephylo) (Jombart et al., 2010a)

We introduce treespace, an R package providing a comprehensive toolkit for the analysis of phylogenetic incongruence. We generalize a previous approach (Amenta & Klingner, 2002; Hillis et al., 2005) for visualizing relationships between trees in a continuous, low‐dimensional Euclidean space to any tree metric, and implement the most common ones (Table 1). In addition, we provide a range of clustering methods permitting the identification of groups of similar trees commonly known as “*tree islands*” (Maddison, 1991) and implement a new method for defining summary trees (Kendall & Colijn, 2016). Our R package also implements a user‐friendly web interface giving access to all of the package's features and permitting the interactive visualization and analysis of sets of phylogenetic trees. To maximize data interoperability, it is fully integrated alongside existing standards for phylogenetics (Jombart, Balloux, & Dray, 2010; Popescu et al., 2012; Schliep, 2011) in the r software (R Core Team 2016).

## 2 IMPLEMENTED METHODS

treespace generalizes an approach used by Amenta and Klingner (Amenta & Klingner, 2002) and later by Hillis et al. (2005), implemented as the treesetviz module for mesquite (Maddison & Maddison, 2003). This method used the Robinson–Foulds metric (Robinson & Foulds, 1979, 1981) to visualize relationships between labelled trees with identical tips in a Euclidean space. Here, we generalize this approach to any tree metric, and add the use of multiple clustering approaches to formally identify “tree islands”.

**Figure 1:**
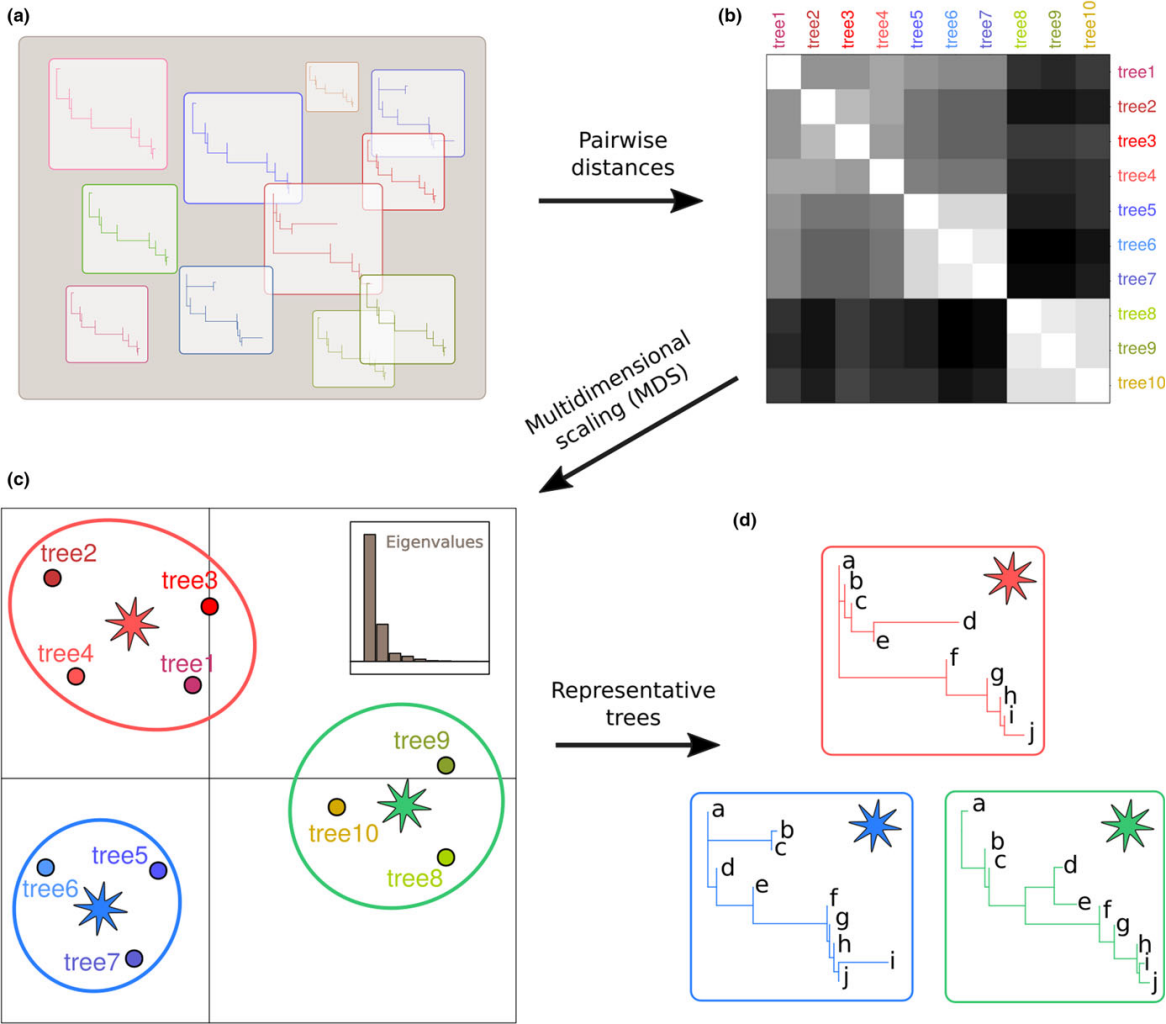
Rationale of the approach used in treespace. This diagram illustrates the four‐step approach for exploring phylogenetic tree spaces in treespace. (a). The input is a set of rooted, labelled trees describing the same taxa. Colours are used here to represent variability amongst trees. (b). Pairwise Euclidean distances between trees are computed, using various tree “summaries” or metrics. (c). These distances are represented in a space of lower dimension using multidimensional scaling (MDS), and potential groups of similar trees can be identified using various clustering methods. (d)**.** Representative trees are derived from each group [Colour figure can be viewed at wileyonlinelibrary.com]

The core idea underlying tree space exploration is to map variability in tree topology or branch length onto a low‐dimensional, Euclidean space, which can then be used for visualizing relationships between the phylogenies and, potentially, to define clusters of similar trees (Figure 1). First, pairwise distances between all pairs of trees in the sample are computed (Figure 1a,b). Typically, measures of distances between trees rely on mapping each phylogeny to a vector of labelled numbers corresponding to pairwise comparisons of tips or internal nodes and then computing the Euclidean distance between the resulting vectors (Figure S1). treespace implements an extensive selection of distances relying on this principle (Kendall & Colijn, 2015; Pavoine et al., 2008; Robinson & Foulds, 1979, 1981; Steel & Penny, 1993; Williams & Clifford, 1971), as well as the BHV metric (Billera, Holmes, & Vogtmann, 2001), which directly computes distances between trees without intermediate feature extraction (Table 1).

Once pairwise distances between trees are computed, they are decomposed into a low‐dimensional space using metric multidimensional scaling (MDS), also known as principal coordinate analysis (PCoA, Gower, 1966; Dray & Dufour, 2007; Legendre & Legendre, 2012). This method finds independent (uncorrelated) synthetic variables, the “principal components” (PCs), which represent as well as possible the original distances inside a lower‐dimensional space (Figure 1c). By inspecting the proportion of the total distances between trees represented by specific axes (the “eigenvalues” of the different PCs), one can assess the number of relevant PCs to examine and, ideally, separate structured phylogenetic variation from random noise (Legendre & Legendre, 2012). Importantly, MDS can only be applied to Euclidean distances (Legendre & Legendre, 2012). In the case of non‐Euclidean tree distances (Billera et al., 2001; Robinson & Foulds, 1981), we use Cailliez's transformation (Cailliez, 1983) to render these distances Euclidean before MDS.

Exploring tree spaces using MDS allows the main features of a given phylogenetic landscape to be explored and evaluated. In particular, the resulting typology may exhibit discrete clusters of related trees (the “phylogenetic islands”), indicating that several distinct phylogenies may actually be supported by the data (Figure 1c). To identify such clusters formally, we implemented various hierarchical clustering methods based on the projected distances, including the single linkage, complete linkage, Unweighted Pair Group Method with Arithmetic Mean (UPGMA) and Ward's method (Legendre & Legendre, 2012).

This approach allows the user to seek representative trees for each cluster separately (Figure 1d). A method for selecting such representative trees is given in Kendall and Colijn (2015) and implemented in treespace as the function “medTree.” This function identifies the geometric median tree(s), which are the tree(s) closest to the mean of the Kendall–Colijn tree vectors for a given cluster. Such trees serve as alternatives to other summary tree approaches such as the consensus tree (Felsenstein, 1985) or the maximum clade credibility (MCC) tree (Drummond & Rambaut, 2007; Ronquist & Huelsenbeck, 2003), with the key advantage that they correspond to specific trees in the sample, thus avoiding implausible negative branch lengths (Heled & Bouckaert, 2013). However, given a collection of trees in a cluster, any summary approach such as MCC could be used.

All the functionalities described above are implemented in treespace as standard R functions, fully documented in a vignette tutorial, as well as in a user‐friendly web interface for interactive data analysis. This interface can be started locally (i.e. without Internet connection) from R using a simple instruction (treespaceServer()) and, therefore, demands virtually no knowledge of the R language. Alternatively, we also provide an online instance of the application at http://shiny.imperial-stats-experimental.co.uk/users/mlkendal/treespace

## 3 WORKED EXAMPLE

**Figure 2:**
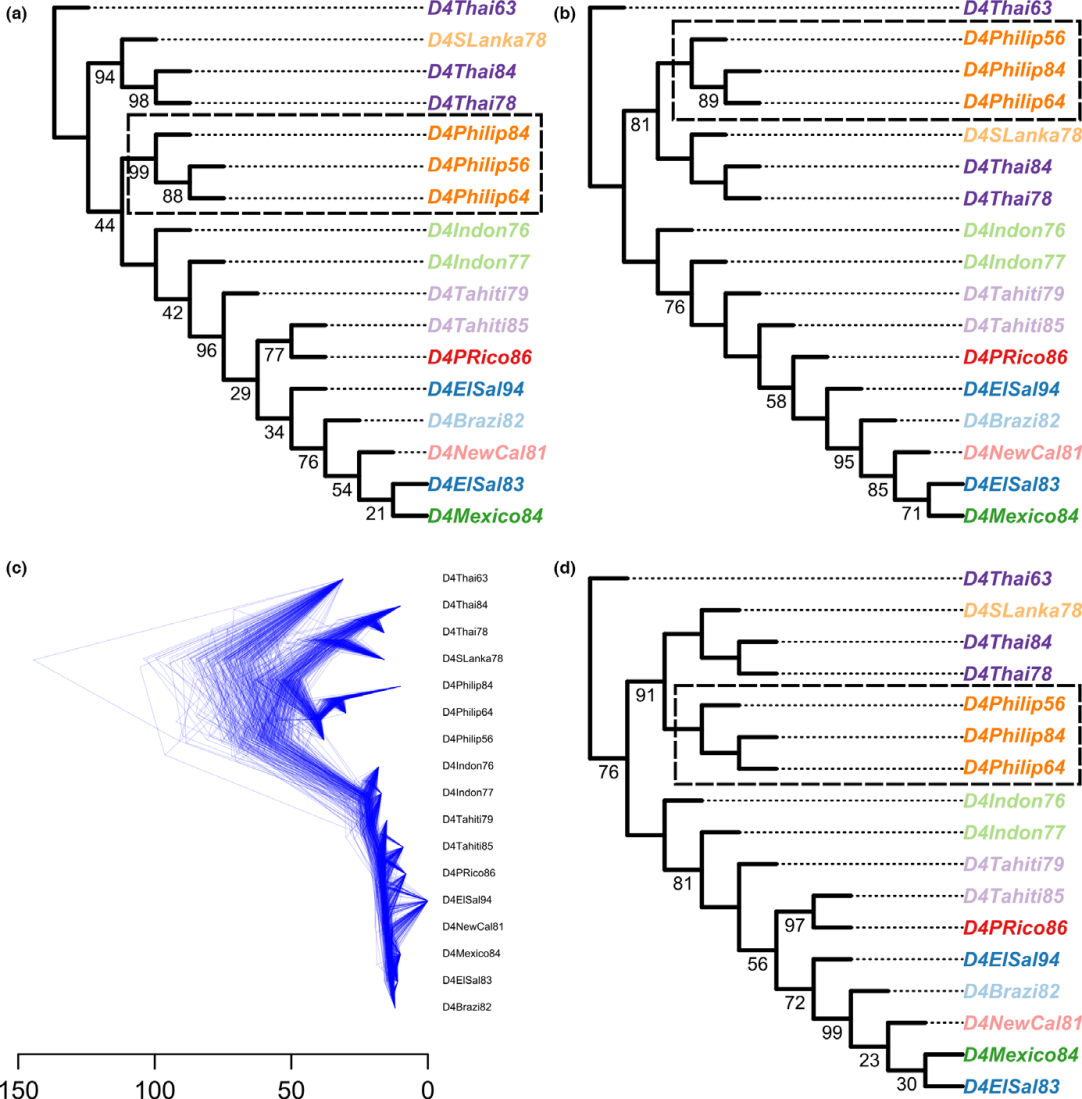
Dengue virus phylogenies obtained by various inference methods, demonstrating the variety of results. (a) neighbour‐joining (NJ), (b) maximum‐likelihood (ML), (c,d) beast, where (c) is a densitree plot of 200 trees randomly sampled from the converged beast posterior, and (d) is the MCC tree from this sample. Bootstrap support values for NJ and ML trees and posterior support values for the beast MCC tree were calculated; values below 100% are shown. The dashed lines delineate the Philippines clade, referred to in the text [Colour figure can be viewed at wileyonlinelibrary.com]

As an illustration, we used treespace to analyse 17 publicly available sequences of dengue virus (Drummond & Rambaut, 2007; Lanciotti, Gubler, & Trent, 1997). This analysis is reproduced in a vignette distributed with the package which can be loaded using the instruction vignette(“DengueVignette”). Three types of phylogenetic trees were obtained: (a) a neighbour‐joining (NJ) tree (Figure 2a) created using the R package ape (Paradis et al., 2004); (b) a maximum‐likelihood (ML) tree (Figure 2b) obtained using phangorn (Schliep, 2011); and (c) Bayesian trees using beast v1.8 with the codon‐position‐specific substitution model and relaxed clock priors, as specified in xml file S2 in (Drummond & Rambaut, 2007). 100 bootstrap trees were obtained for the NJ and ML phylogenies (Holmes, 2003a). For beast, 200 trees were randomly sampled from the posterior distribution after visually assessing the convergence of the MCMC chain with 10,000,000 iterations. Results were qualitatively unchanged using larger samples. The NJ and ML trees were rooted using the *“D4Thai63”* sequence, seen as the most basal in the beast MCC tree.

Trees inferred using the three methods were different (Figure 2) in the position of the “Philippines clade” (dashed box in Figure 2) and in whether the *Tahiti84* tip was sister to *PRico86*. Bootstrap support values for the NJ tree show considerable phylogenetic incongruence, both near the tips and deep in the tree (Figure 2a). In contrast, the ML tree has high bootstrap support for most nodes (Figure 2b). Interestingly, the ML and NJ trees themselves were quite different (Figure 2a,b), notably with the “Philippines” clade clustered with isolates from Thailand and Sri Lanka (“*D4Thai”* and “*D4SLanka”* isolates) in the ML tree and not in the NJ phylogeny. Examination of bootstrap values alone does not indicate whether the NJ and ML bootstrap trees exhibit any common topologies. beast trees visualized using densitree (Bouckaert, 2010) and the beast MCC tree (Figure 2c,d) seemed more similar to the ML phylogeny in the position of the “Philippines” clade, but also showed uncertainty in tree topologies in multiple places. While densitree plots provide intuition about the extent of incongruence amongst these trees, Figure 2c does not reveal whether the topologies of beast phylogenies coincide with any of the other trees.

**Figure 3:**
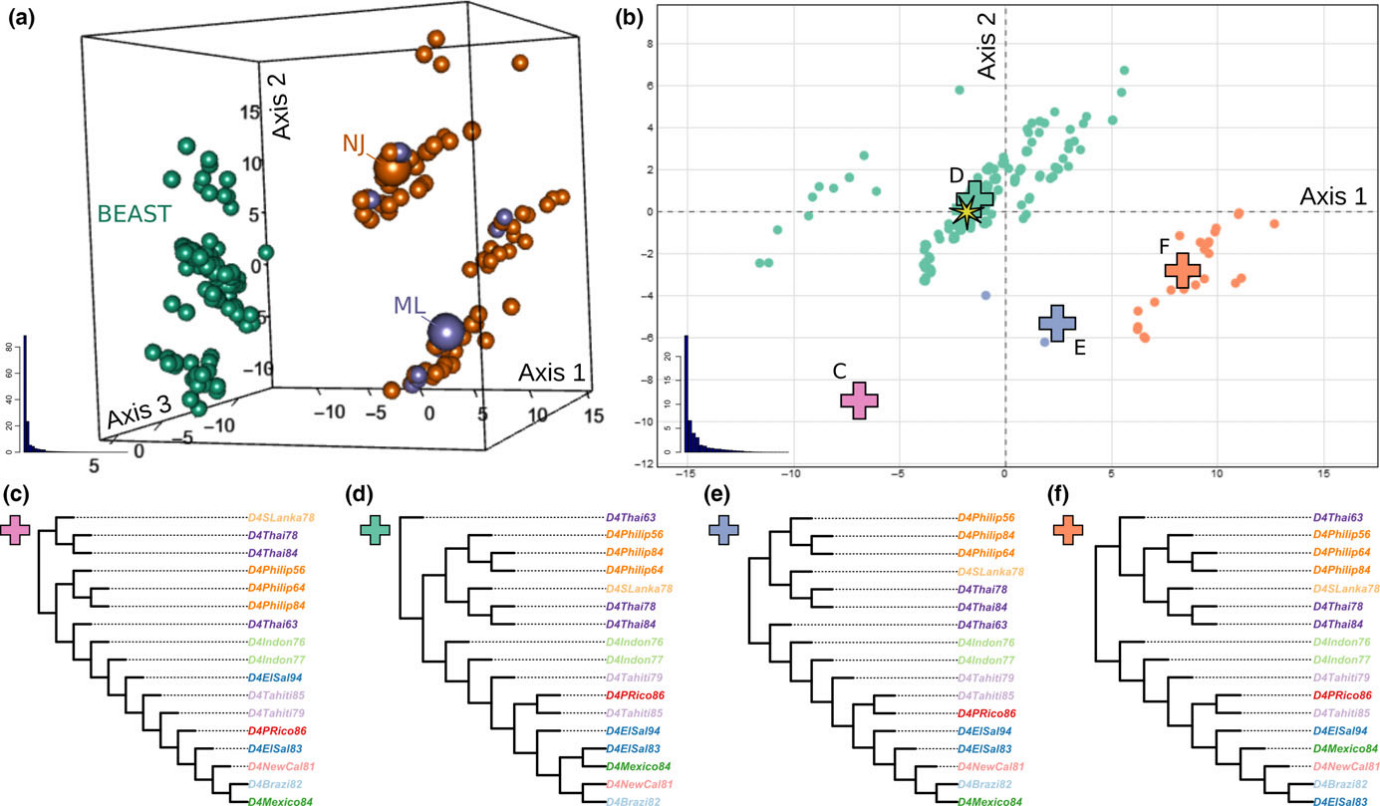
An analysis of the dengue virus phylogenies from figure 2 using treespace. (a) Three‐dimensional MDS plot demonstrating the variety between phylogenies inferred by different methods. The NJ and ML trees are indicated by larger spheres, with their corresponding bootstrap trees marked as smaller spheres of the same colour. (b) Two‐dimensional MDS plot of the beast trees alone, coloured by cluster obtained using the function findGroves. Scree plots are given as insets. (c–f) From each cluster in (b), a median tree was selected using medTree. These are highlighted in (b) by crosses. The MCC tree (Figure 2d) is indicated by a star in (b), and sits close to the green median tree (d). Indeed, these two trees differ only in their topologies amongst the tips “*D4Brazil82,*” “*D4NewCal81,*” “*D4Mexico84*” and “*D4ElSal83*” [Colour figure can be viewed at wileyonlinelibrary.com]

We used treespace to investigate potential discrepancies in more detail. A three‐dimensional MDS based on the Kendall–Colijn metric (Kendall & Colijn, 2015) revealed differences between the different methods (Figure 3a; see vignette for an interactive version). This analysis revealed that topologies of NJ and ML bootstrap trees were broadly similar, overlapping in three distinct and similar‐sized clusters. However, the NJ trees exhibited slightly more variation, including a few outlying topologies (top right, Figure 3a), which is consistent with the overall lower bootstrap support values than in the ML tree (Figure 2).

beast trees formed a group of their own, with no overlap between their topologies and those of the NJ or ML trees (Figure 3a). A separate analysis of the beast trees revealed four distinct clusters of topologies (function “findGroves,” Figure 3b). Closer examination of the phylogenies revealed that topologies of these sets of trees were indeed all different; no single topology was shared between beast trees and NJ/ML trees. The median trees (function “medTree”) obtained for each cluster (Figure 3c–f) revealed that Bayesian trees largely supported the positioning of the “Philippines” clade of the ML tree (Figure 3d,f), with alternative placements mostly due to a few outlying topologies more akin to the NJ tree (Figure 3c,e). These results also suggested that the position of root may be disputed, as every phylogenetic islands exhibited a different rooting.

## 4 DISCUSSION

treespace provides a simple framework for exploring landscapes of phylogenetic trees and investigating phylogenetic incongruence using tree–tree distances. Of the various methods for measuring distances between trees, some may be better than others at capturing meaningful topological differences, as is the case when testing phylogenetic signal (Jombart, Pavoine, Devillard, & Pontier, 2010; Münkemüller et al., 2012; Pavoine et al., 2008). There are currently no theoretical descriptions that can determine a priori which tree comparison method will be most revealing for which kind of data. Recognizing this, we have incorporated considerable flexibility into treespace in terms of how trees are compared, by providing a framework which can incorporate any tree‐to‐tree distance, and implementing seven different ones by default. This feature distinguishes treespace from other similar software, like the R package RWTY which re‐implements mesquite's treesetviz module (Robinson–Foulds metric) as part of an excellent toolkit for assessing mixing in Bayesian phylogenetics (Warren, Geneva, & Lanfear, 2017), or treescaper, which puts stronger emphasis on reduced space optimization methods and community detection algorithms (Huang et al., 2016; Wilgenbusch et al., 2017).

treespace combines a fast dimension reduction technique (MDS) with various hierarchical clustering approaches (Legendre & Legendre, 2012) to reveal phylogenetic tree islands. While this approach is very computer‐efficient, it may sometimes struggle to delineate tree islands in the presence of distortions of the tree space observed in some specific metrics (Hillis et al., 2005). For instance, recent work suggests that the Robinson–Foulds metric is best combined with nonlinear dimension reduction techniques for identifying clusters of similar trees (Wilgenbusch et al., 2017). Further efforts should be devoted to investigating alternative dimension reduction approaches such as the t‐SNE implemented with a Barnes–Hut approximation (van der Maaten & Hinton, 2008), and nonlinear classifiers such as support vector machines (Schölkopf & Smola, 2002) or community detection methods (Blondel, Guillaume, Lambiotte, & Lefebvre, 2008; Huang et al., 2016).

Our approach is very different from the “principal component analysis” (PCA) for trees introduced by Aydin, Pataki, Wang, Bullitt, and Marron (2009) and extended to phylogenetic trees by Nye (2011). These methods proceed by analogy to classical PCA (Hotelling, 1933; Pearson, 1901), but do not actually map trees into vector spaces, and are therefore unable to use classical dimension reduction techniques and the corresponding visualizations (Legendre & Legendre, 2012). They produce optimal “tree lines” (Aydin et al., 2009), which are collections of nested trees meant to be representative of the entire tree set. While this concept is undoubtedly interesting, it does not provide a direct geometric representation for the trees, so that it cannot be used to assess relationships between the different phylogenies or identify phylogenetic islands (Maddison, 1991). In fact, while conceptually different, the identification of clusters of trees implemented in treespace is related to the idea of boundaries between tree topologies (Holmes, 2003b), and to the notion of “terraces” in the phylogenetic tree space (Sanderson, McMahon, & Steel, 2011). Both “boundaries” and “terraces” define regions of the tree space inside which trees are closely related through their topology (Holmes, 2003b; Sanderson et al., 2011) and their log‐likelihood under a specific evolutionary model (Sanderson et al., 2011). While we do not currently include the latter, it would be interesting to incorporate information on tree log‐likelihood as weights in the analysis.

Lastly, one of the key advantages of developing treespace within the R software (R Core Team 2016) is the resulting interoperability with other tools. Indeed, R is becoming a standard for phylogenetic analyses (Jombart et al., 2010, 2017; Kembel et al., 2010; Paradis et al., 2004; Revell, 2012; Schliep, 2011; Warren et al., 2017) and therefore represents an ideal environment for treespace to become a useful tool for the exploration of phylogenetic results. Its development within an open‐source, community‐based platform together with its availability as user‐friendly web interface will hopefully facilitate its adoption by a wide range of scientists and encourage further methodological developments.

## AUTHOR CONTRIBUTION

TJ, MK and JAG developed the package treespace. MK collated and analysed the data. TJ, MK, JAG and CC contributed to writing the manuscript.

## SOFTWARE AVAILABILITY

The stable version of treespace is released on the Comprehensive R Archive Network (CRAN): http://cran.r-project.org/web/packages/trescape/index.html and can be installed in R by typing: install.packages(“treespace”). The development version of treespace is hosted on github: https://github.com/thibautjombart/treespace and can be installed in R using the devtools package by typing: devtools::install_github(“thibautjombart/treespace”). treespace is distributed under GNU Private Licence (GPL) version 2 or greater. It is fully documented in a vignette accessible by typing: vignette(“treespace”). treespace is documented in a dedicated website: https://thibautjombart.github.io/treespace/.
