# Supplementary figures and images for: treespace: Statistical exploration of landscapes of phylogenetic trees
Source: Mol Ecol Resour. 2017 May 15;17(6):1385–92. doi: 10.1111/1755-0998.12676 (PMC5724650; doi:10.1111/1755-0998.12676)

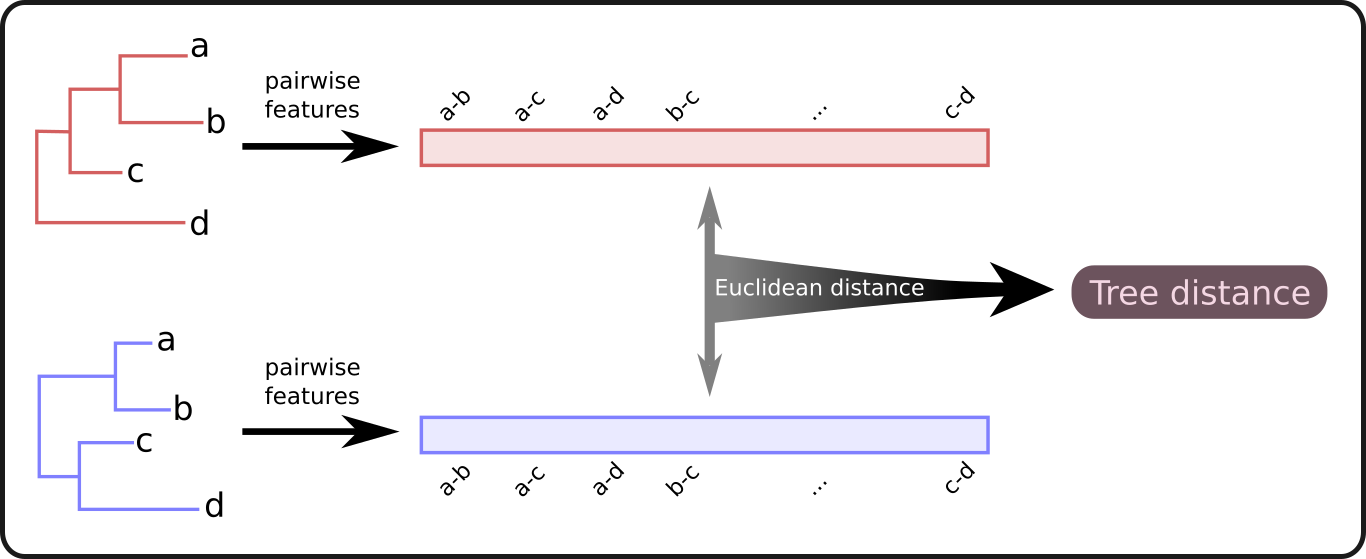

Supplement: Supplementary file 1 [file MEN-17-1385-s001.png]
